# Interaction Between Object-Based Attention and Pertinence Values Shapes the Attentional Priority Map of a Multielement Display

**DOI:** 10.1037/xhp0000194

**Published:** 2016-01-11

**Authors:** Celine R. Gillebert, Anders Petersen, Chayenne Van Meel, Tanja Müller, Alexandra McIntyre, Johan Wagemans, Glyn W. Humphreys

**Affiliations:** 1 Department of Experimental Psychology, University of Oxford; 2 Department of Psychology, University of Copenhagen; 3 Department of Experimental Psychology, University of Oxford; 4 Department of Brain & Cognition, University of Leuven (KU Leuven); 5 Department of Experimental Psychology, University of Oxford

**Keywords:** perceptual organization, visual selection, visual short-term memory, attentional priority map, Theory of Visual Attention

## Abstract

Previous studies have shown that the perceptual organization of the visual scene constrains the deployment of attention. Here we investigated how the organization of multiple elements into larger configurations alters their attentional weight, depending on the “pertinence” or behavioral importance of the elements’ features. We assessed object-based effects on distinct aspects of the attentional priority map: top-down control, reflecting the tendency to encode targets rather than distracters, and the spatial distribution of attention weights across the visual scene, reflecting the tendency to report elements belonging to the same rather than different objects. In 2 experiments participants had to report the letters in briefly presented displays containing 8 letters and digits, in which pairs of characters could be connected with a line. Quantitative estimates of top-down control were obtained using Bundesen’s Theory of Visual Attention (1990). The spatial distribution of attention weights was assessed using the “paired response index” (PRI), indicating responses for within-object pairs of letters. In Experiment 1, grouping along the task-relevant dimension (targets with targets and distracters with distracters) increased top-down control and enhanced the PRI; in contrast, task-irrelevant grouping (targets with distracters) did not affect performance. In Experiment 2, we disentangled the effect of target-target and distracter-distracter grouping: Pairwise grouping of distracters enhanced top-down control whereas pairwise grouping of targets changed the PRI. We conclude that object-based perceptual representations interact with pertinence values (of the elements’ features and location) in the computation of attention weights, thereby creating a widespread pattern of attentional facilitation across the visual scene.

To create an efficient representation of the world, our brain continuously selects a fraction of the information that reaches our senses and organizes it into coherent and meaningful objects. The research supporting object-based attentional selection was inspired by the observation that visual perception depends on a series of grouping principles first articulated by the Gestalt psychologists in the early 20th century (Wertheimer, 1923; for a recent review, see Wagemans et al., 2012). For example, we tend to group objects that are close to one another (Gestalt law of proximity), have similar properties (Gestalt law of similarity), or are connected to each other (Gestalt law of connectedness). In the remainder of the article, we will refer to elements organized by one or more Gestalt laws and/or uniform connectedness as a perceptual “object” (Kimchi, Yeshurun, & Cohen-Savransky, 2007).

Evidence for the hypothesis that objects constrain the deployment of attention has been obtained in several paradigms with both healthy participants and patients with brain lesions. In divided attention paradigms, it is easier to make perceptual judgments about two targets when they group to form an object compared with when they occur on different objects (Behrmann, Zemel, & Mozer, 1998; Duncan, 1984; Lavie & Driver, 1996; Vecera & Farah, 1994). In focused attention paradigms, distracters are more likely to impair performance when they are grouped with a target by factors such as connectedness, color, similarity, or good continuation (Baylis & Driver, 1992; Driver & Baylis, 1989; Harms & Bundesen, 1983; Kahneman & Henik, 1981; Kramer & Jacobson, 1991). Similarly, response times (RTs) in visual search tasks are longer when distracters resemble and group with the target (Duncan & Humphreys, 1989), but shorter with increased similarity between distracters (Humphreys, Quinlan, & Riddoch, 1989).

Several accounts have been proposed to explain object-based effects in these different paradigms. The two most often put forward are the attentional spreading and attentional prioritization accounts, respectively. The “attentional spreading” or “sensory enhancement” account posits that attention *automatically* spreads through an attended perceptual group, thereby improving the rate and efficiency of perceptual processing of the attended relative to unattended objects (e.g., Chen & Cave, 2008; Richard, Lee, & Vecera, 2008; Vecera & Farah, 1994). Similarly, low attentional weights could spread through an object when all object elements are task-irrelevant, thereby facilitating their suppression (Dent, Humphreys, & Braithwaite, 2011). In contrast, the “attentional prioritization” or “search prioritization” hypothesis (Shomstein & Behrmann, 2008; Shomstein & Yantis, 2002, 2004) posits that there is a tendency to assign higher attentional weights to spatial locations within an already attended object compared with spatial locations in other objects (as also predicted by the attentional spreading account), but only when the location of an upcoming target is uncertain. When certain about the spatial location of the target, a high priority should be assigned only to the target location and not to the other locations on the same and other objects (i.e., there is no need to search the target)—thereby eliminating object-based effects.

Object-based effects not only depend on the presence of predictive factors (e.g., about the target location), but also on the extent of attentional focus (Goldsmith & Yeari, 2003; Lavie & Driver, 1996; Shomstein & Yantis, 2002) and the goodness of the object representation (e.g., Chen & Cave, 2008; Law & Abrams, 2002), among other factors (see Chen, 2012 for a review). These influential factors suggest that attention tends to spread within an object, as predicted both by the attentional spreading and the attentional prioritization accounts, but that the spreading of attention is not necessarily automatic (Chen, 2012; Freeman, Macaluso, Rees, & Driver, 2014), as proposed by the latter account only.

A somewhat different approach is to conceive object-based effects within biased-competition accounts of attention, which propose that selection of perceptually salient or behaviorally relevant information can be completed via several interacting mechanisms—including the strength of grouping between elements but also the strength of top-down attention to the stimulus (Desimone & Duncan, 1995; Duncan, 2006). Top-down attention can selectively be allocated to objects (Duncan, 1984; Egly, Driver, & Rafal, 1994; Vecera & Farah, 1994), but also to spatial locations (Posner, 1980; Posner, Cohen, & Rafal, 1982) or to features (e.g., color) (e.g., Baylis & Driver, 1992; Driver & Baylis, 1989; Harms & Bundesen, 1983; Kramer & Jacobson, 1991). “Pertinence values” are used to weigh these different sources of attentional guidance (object-based, space-based, and feature-based) depending on their behavioral importance, and as such determine which elements in the environment will be preferentially processed (Desimone & Duncan, 1995). The resulting pattern of attentional facilitation can be conceptualized by means of an “attentional priority map,” a topographical representation of the environment where each location is weighed depending on sensory evidence, expectations and internal goals (Bisley & Goldberg, 2010; Bundesen, 1990; Bundesen, Habekost, & Kyllingsbæk, 2005; Itti & Koch, 2000; Molenberghs, Gillebert, Peeters, & Vandenberghe, 2008; Ptak, 2012).

Despite being central to the biased-competition accounts of attention, few studies have explicitly addressed the interaction within and between different types of perceptual representations in the computation of attentional weights. In one study, Kravitz and Behrmann (2008) showed that cueing a location within an object not only facilitates the perception of a target presented within the same object (object-based attention) but also at locations in the space surrounding the object (space-based attention). In a different study, the same authors showed that object-based effects (better performance for targets appearing in a cued compared with an uncued object) are reduced with increased featural similarity between the cued and uncued object. Furthermore, perceptual or semantic similarity between the cued and uncued object also facilitates performance in the space surrounding the uncued object (Kravitz & Behrmann, 2011). However, how embedding elements into larger objects affects the spatial distribution of attentional weights when attention is not cued to a particular object, has not been studied. Furthermore, it remains unclear whether object-based representations affect attentional weights in a similar way depending on the behavioral importance or pertinence values of the elements’ features.

Noteworthy, all of the experiments mentioned above used displays containing one or two objects, where participants had to identify either up to two targets (in divided attention paradigms) or one target in the presence of distracters (in focused attention paradigms). In real life, however, a visual scene typically contains several elements: some of them are embedded in larger perceptual groups and others are not, and some that are important for us to select, and others that are not. This raises the question as to whether object-based representations modulate the computation of attentional weights in conditions with a high perceptual load, for example, when the number of elements in the visual field exceeds the capacity of visual short-term memory (VSTM; typically between 3 and 4 objects, Cowan, 2001; Luck & Vogel, 1997) such that attentional selection is critical for successful task performance.

The aim of the current study was to formally assess how object-based representations interact with pertinence values (of the elements’ features and spatial location) to shape the attentional priority map of a multielement display. We addressed this question within the framework of the Theory of Visual Attention (TVA), which provides probably the best quantitative account to date for attentional selection from multielement displays (Bundesen, 1990).

TVA is a mathematical formalization of the biased competition account (Desimone & Duncan, 1995), that allows quantitative estimation of several attentional parameters. For selection from multielement displays, TVA is a mathematical derivation of the fixed-capacity independent race model (FIRM; Shibuya & Bundesen, 1988). Within TVA, attentional selection is defined as a parallel race between competing elements for access to VSTM with limited storage, where the competition is influenced by bottom-up and top-down factors. According to TVA, the processing of a multielement display consists of two waves. During the first wave of processing, an attentional weight is computed for each element in the display, reflecting the sensory evidence that the element is perceptually salient or behaviorally important. More specifically, the attentional weight of an object *x* (ω_*x*_), is a weighted sum of pertinence values, where the importance (“pertinence”) of a given category (e.g., a feature or a spatial location) is weighted by the strength of the sensory evidence that the object belongs to the category. Pertinence values (also referred to as priority values) reflect the current importance of attending to elements belonging to a certain category, and as such determine which objects are selected (a process known as “filtering”). The efficiency of top-down control is expressed in the parameter α, which equals the ratio between the attentional weight of a distracter and a target. If top-down control is efficient, attentional weights for targets will be higher than for distracters. The second wave of processing is the race between the elements for access to VSTM. The total processing capacity at this stage is given by a constant, *C* (*processing speed* in elements/second), which is distributed across the elements in the display according to their relative weights. The processing capacity allocated to the element determines the time needed to access VSTM. According to TVA, the encoding times for competing elements are stochastically independent, and only the first elements that finish processing before *K* (*capacity of VSTM* in elements) is reached or before the stimulus presentation terminates, are stored in VSTM and are accessible for explicit report.

TVA-based assessment is typically done through simple letter identification tasks. According to TVA, the probability of reporting *n* letters when the presentation time is larger than *t*_*0*_ (minimum effective exposure duration) can be captured by a psychometric function that varies between individuals (see Figure 2, for an example). The function can be obtained by combining a “whole report” task (Sperling, 1960), in which an array of letters is briefly presented and participants are asked to verbally report the identity of as many letters as they can, with a “partial report” task (Shibuya & Bundesen, 1988), where participants are asked to report the identity of only a subset of the stimuli, for example, only the letters printed in a certain color. The number of correctly reported letters is analyzed as a function of exposure duration. The resulting exponential function can best be described by five independent components: (a) *t*_*0*_, the threshold of conscious perception (the longest ineffective exposure duration); (b) *K,* VSTM capacity; (c) *C*, processing speed; (d) α, top-down control; and (e) ω-values determining the spatial distribution of attentional weights. Here we used a variant of the whole/partial report task, combining features of a divided and a selective attention task, to investigate object-based effects on two components of the attentional priority map, top-down (feature-based) control and the spatial distribution of attentional weights.

We hypothesized that pairwise grouping of elements in a multielement display would modulate top-down control (parameter α) depending on the (feature-based) pertinence values of the elements belonging to the same object. In particular, we predicted that pairwise grouping of elements along the task-relevant dimension (targets with targets, distracters with distracters) would increase top-down control, as high attentional weights would spread in the target-related objects and low attentional weights would spread in the distracter-related objects. In contrast, we hypothesized reduced top-down control when each target would be grouped with a distracter.

In addition, we posited that the organization of elements in several larger configurations would change the spatial distribution of the attention priority map by altering the pertinence values of the spatial locations. In particular, we expected attentional weights to be evenly distributed across the individual target elements for displays without grouping, but concentrated on a few spatial locations when target elements were pairwise grouped into larger objects. In other words, we predicted that similar attention weights would be assigned to elements that belong to the same object compared with different objects. Noteworthy, when behavioral importance is determined based on the elements’ features, the pertinence values of spatial locations are independent from the differentiation in attentional weights between targets and distracters.

For the purpose of this study, objects were created by pairwise grouping the elements using the principle of uniform connectedness. Previous studies showed that the reliability of object-based effects on attention depends on the “goodness” of the objects. For instance, objects with closed boundaries (e.g., Marino & Scholl, 2005), showing uniform connectedness (e.g., Watson & Kramer, 1999) and with elements appearing on the same straight line within an object (Crundall, Cole, & Galpin, 2007) are more likely to induce a same-object bias. To maximize the likelihood of finding object-based effects in our study, targets and/or distracters were grouped using the principle of uniform connectedness, and the elements appeared on the same line within each object.

## General Method

### Participants

We conducted two behavioral experiments using a total of 44 healthy volunteers. All participants were strictly right-handed, reported normal or corrected-to-normal vision, were free of psychotropic and vasoactive medication, and had no neurological or psychiatric history. A participant who took part in Experiment 1 was excluded because of technical problems during data acquisition. We had 21 participants in the first experiment (11 women, aged 18–25 years), and 22 (14 women, aged 22–40 years) in the second experiment.

### Materials and Procedure

#### Apparatus

The stimuli were created in MATLAB 2012 (The MathWorks, Inc., Natick, MA) and presented using the Psychtoolbox v.3.0 package for MATLAB (Kleiner et al., 2007). The stimuli were displayed on a 24-in. LED monitor (ViewSonic V3D245, ViewSonic North America, Brea, CA) with a spatial resolution of 1,920 × 1,080 pixels and a refresh rate of 100 Hz, placed 57 cm in front of the participant. Responses were collected via the keyboard.

#### Stimuli and procedure

**Figure 1 fig1:**
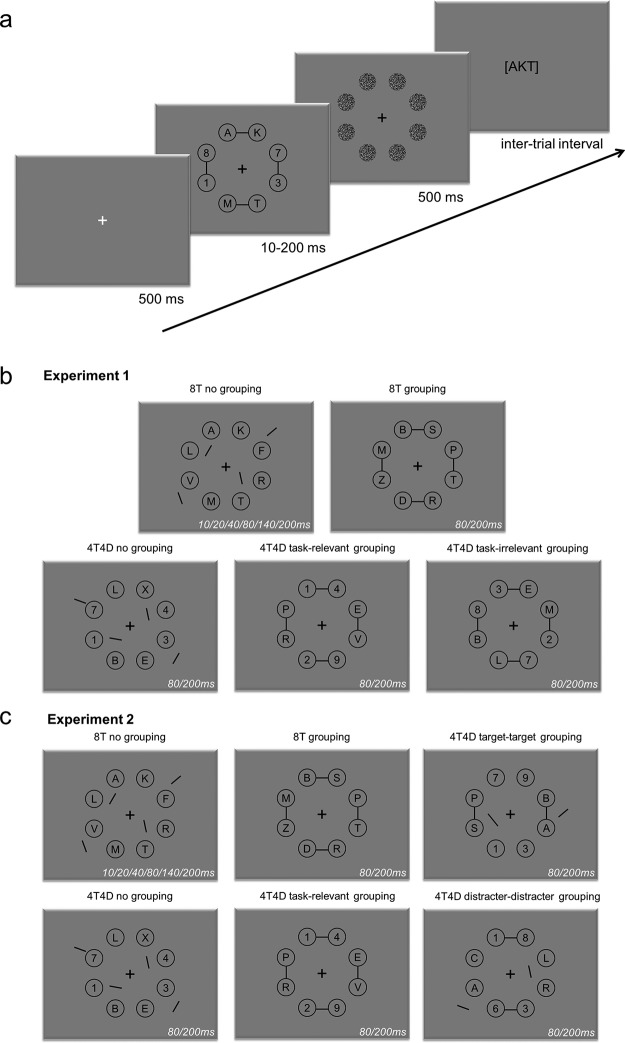
Experimental paradigm. (a) Outline of a single trial showing the timing of the different events. (b) Experiment 1: Illustration of the five different display types used: eight target letters not grouped or grouped by connectedness (top row), four targets intermixed with four distracters either not grouped, or grouped according to task relevance, or each target grouped with a distracter (bottom row). (c) Experiment 2: Illustration of the six different display types used: eight target letters not grouped or grouped by connectedness (top row, first two columns), four targets intermixed with four distracters either not grouped, or grouped according to task relevance (bottom row, first two columns), four targets intermixed with four distracters where either the targets are grouped (top row, last column) or the distracters are grouped (bottom row, last column).

We designed a variant of the “CombiTVA” paradigm (Vangkilde, Bundesen, & Coull, 2011), which combines two classic paradigms: the whole report paradigm (Sperling, 1960) where all stimuli are to be reported, and the partial report paradigm (Shibuya & Bundesen, 1988) in which only stimuli with a certain target feature must be reported. For the purpose of this study, target stimuli were letters drawn from the set [A,B,D,E,F,G,H,J,K,L,M,N,P,R,S,T,V,X,Z], and distracters were digits drawn from the set [1,2,3,4,5,6,7,8,9]. We presented eight target stimuli in whole report trials, and four target stimuli intermixed with four distracter stimuli in partial report trials. All trials followed the same basic design outlined in Figure 1a. A trial was initiated by a white fixation cross (size 0.5°) presented for 500 ms against a gray background. This was followed by the stimulus display, containing eight black characters (height 0.8°, width 0.6°) presented on an imaginary circle (*r* = 4° of visual angle) around a black fixation cross. Each character was presented in the center of a black circle outline (diameter 1.5°). Depending on the experimental condition (see Figure 1b for examples), pairs of circles were connected (“grouped”) by a black line (approximately 0.1° thick) along the horizontal or the vertical axis. In the other trials, the four black lines were pseudorandomly oriented and presented separately, two of them being placed inside the stimulus configuration (one in the left, one in the right hemifield) and two outside the configuration (one in the left, one in the right hemifield). The distance between the fixation point and the lines was on average the same as in the grouping conditions. The stimulus display was presented for 10 to 200 ms and terminated by a 500 ms masking display containing eight circular random-noise patterns (size 1.5°). The masks were presented to erase the visual afterimage and precisely control the effective exposure duration of the stimuli.[Fig-anchor fig1]

Participants were instructed to fixate centrally at all times, and to make a nonspeeded report of the identity of all target letters they were “fairly certain” of having seen. They were informed of the accuracy of their reports (the number of correctly reported letters divided by the number of reported letters) after each block and were encouraged to keep their reports within a specified accuracy range of 80–90% correct.

## Experiment 1: Basic Effects of Grouping

In this experiment, we investigated the effect of task-relevant (targets with targets, distracters with distracters) and task-irrelevant (targets with distracters) grouping on the attentional priority map. We tested the hypothesis that task-relevant grouping would increase top-down control for targets compared with distracters, and that task-irrelevant grouping would decrease this top-down control. In addition, we also tested the hypothesis that task-relevant grouping would change the spatial distribution of attentional weights across the entire visual scene.

### Method

#### Experimental conditions

Five different stimulus displays were realized, with either only targets being presented (8T, whole report) or targets and distracters being presented (4T4D, partial report; Figure 1b). In the 8T no grouping condition, we presented eight ungrouped target letters at exposure durations varying between 10 and 200 ms (10/20/40/80/140/200 ms). The full range of exposure durations for this condition was implemented to estimate VSTM capacity (*K*), perceptual threshold (*t*_*0*_), and processing speed (*C*). In the remaining conditions, we only used two exposure durations (80, 200 ms), which is sufficient to reliably estimate α-parameters once *K* is known (see Kyllingsbæk, 2006). In the 8T grouping condition, the eight targets were pairwise grouped along the horizontal and vertical axis. In the partial report trials, the eight characters were either ungrouped (4T4D no grouping), grouped according to task relevance (4T4D task-relevant grouping: targets grouped with targets, distracters with distracters), or each target was grouped with a distracter (4T4D task-irrelevant grouping). Examples of stimulus displays for the different conditions are depicted in Figure 1b. We collected 60 trials for each of the 16 conditions (Stimulus Display × Exposure Duration) illustrated in Figure 1b. The order of the conditions was randomized for each subject. The task consisted of one practice block of 24 trials, followed by 840 trials, divided into 14 blocks of 60 trials.

#### Data analysis

##### Number of correctly reported letters

The numbers of correctly reported letters in the whole and partial report trials were submitted to repeated measures analysis of variances (ANOVAs) with exposure duration and grouping condition as factors. When sphericity could not be assumed (Mauchly’s sphericity test: *p* < .05), *p* values were adjusted using the Greenhouse-Geisser correction (G–G adj.). Post hoc comparisons included the pairwise comparisons between different task conditions (threshold: *p* < .05, Bonferroni-corrected for multiple comparisons; corrected thresholds whole report trials: *p* < .0125 for the interaction between grouping and exposure duration; corrected thresholds partial report trials: *p* < .0167 for the main effect of grouping condition and *p* < .008 for the interaction between grouping condition and exposure duration).

##### Computational modeling: Analysis on the TVA parameters

**Figure 2 fig2:**
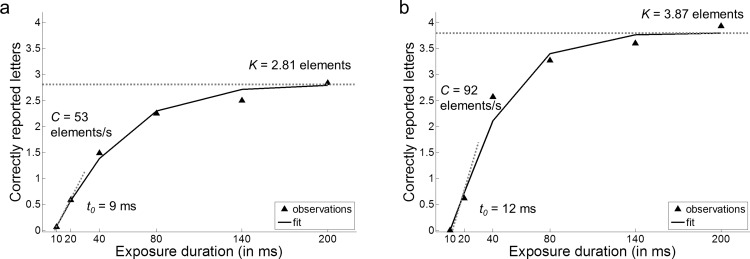
Computational modeling of behavioral data. The number of correctly reported letters in the 8T no grouping conditions from two representative participants illustrating the relationship between the raw data and the TVA-based parameters. In addition to the observed data (black triangles), the scores predicted by the TVA model are plotted (solid black line) to indicate that the model is a good fit to the data and to illustrate how *K* (the asymptotic level of the curve), *t*_*0*_ (the exposure durations at which the curve rises from the abscissa), and *C* parameters (the slope of the curves at *t*_*0*_) are related to these scores. TVA = Theory of Visual Attention.

The number of correctly reported letters in the different conditions was modeled for each participant using a maximum likelihood fitting procedure (Dyrholm, Kyllingsbæk, Espeseth, & Bundesen, 2011; Kyllingsbæk, 2006). For the data in Experiment 1, a model with 6 parameters was used: (a) The capacity of VSTM (*K*^1^; 7 *df*; the asymptotic level of the exponentially increasing function, see Figure 2) reflecting the number of elements that can be maintained in VSTM in parallel; (b) the threshold for conscious perception (*t*_0_; 1 *df*; the starting point of the exponentially increasing function, see Figure 2), measured in milliseconds; (c) the visual processing speed (*C*; 1 *df*; the slope of the exponentially increasing function at *t*_0_, see Figure 2) reflecting the number of elements that can be processed per second; and (d–f) three selectivity parameters (αs; 3 *df*), one for each of the grouping conditions. All parameters were allowed to vary freely, but *t*_0_ was fixed to 0 and the model refitted to the data, if a negative *t*_0_ was found as a result of the initial estimation. The attentional capacity parameters (*K, C*) and the threshold for conscious perception (*t*_0_) were assumed to be common across conditions.[Fig-anchor fig2]

##### Paired response index

To test the hypothesis that object-based representations alter the spatial configuration of the attentional priority map, we examined whether participants were more likely to report two grouped letters as opposed to two separate letters. To this end, we calculated for each condition the average number of correctly reported “pairs” of letters. A pair was defined as two letters presented along the horizontal or vertical axis (i.e., the top two letters, the bottom two letters, the two letters presented to the left, and the two letters presented to the right), the axes along which objects were created in the grouping conditions. The number of correctly reported pairs is inevitably related to the total number of correctly reported letters. To control for this bias, we estimated the number of correctly reported pairs for each participant and each condition, assuming that the correctly reported letters would be randomly distributed across the display. Specifically, for each trial we shuffled the position of the correctly reported letters (across eight possible locations for the whole report trials and across four possible locations for the partial report trials), and recalculated the number of correctly reported pairs for each condition. This process was repeated 10,000 times for each participant. This procedure allowed us to estimate for each participant and each condition the number of correctly reported pairs if the correctly reported letters were randomly distributed across the display. We used these values to calculate the “paired response index” (PRI), by taking the difference between the observed number of pairs and the simulated number of pairs (averaged across 10,000 iterations) for each participant and each condition. This index reflects whether there is a tendency to assign similar attentional weights to elements that belong to the same object compared with different objects. When behavioral importance is determined based on the elements’ features (e.g., color or alphanumerical class), the spatial distribution of attentional weights is independent from the differentiation in attentional weights between targets and distracters.

To assess whether grouping increased the relative frequency of reporting two paired letters, PRIs in the whole and partial report conditions were submitted to a repeated measures ANOVA with exposure duration (80 ms, 200 ms) and grouping as within-subjects factors. The 4T4D task-irrelevant grouping trials (and the corresponding configurations in the 4T4D no grouping condition) were not taken into account for this analysis, as no pairs of letters could be reported in this condition (see Figure 1b).

### Results and Discussion

Using the mean number of correctly reported letters, estimates of *t*_*0*_, *C*, *K*, and αs were obtained through the TVA-based fitting procedure (Tables 1 and 2; see Figure 2, for an example). On average, the TVA-based model was a good fit for the observed data: Across all participants, the average Pearson product–moment correlation coefficient between the observed and predicted scores was 0.98 (*SD*: 0.02); thus, the model explained on average 96% (*r*^2^) of the variance in the data. [Table-anchor tbl1][Table-anchor tbl2]

**Figure 3 fig3:**
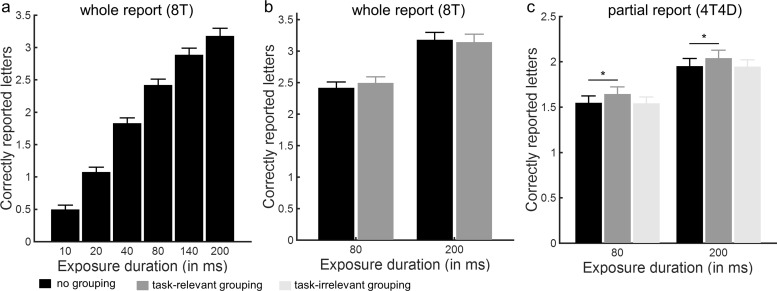
Experiment 1: correctly reported letters. (a) Whole report performance showing the mean number of correctly reported letters as a function of exposure duration in the whole report trials without grouping. Error bars represent *SEM*. (b-c) Mean number of correctly reported letters in the whole report (b) and partial report (c) trials, as a function of exposure duration and grouping condition. Error bars represent *SEM*.

In the whole report trials (Figure 3a and b), a repeated measures ANOVA on the mean number of correctly reported letters with exposure duration (80 ms, 200 ms) and grouping condition (no grouping, task-relevant grouping) as factors revealed a main effect of exposure duration, *F*(1, 20) = 365.59, *p* < .001 and a significant interaction between exposure duration and grouping, *F*(1, 20) = 6.35, *p* = .02, but no main effect of grouping, *F*(1, 20) = .55, *p* = .47. To inform the significant interaction, we compared the grouping conditions at the different exposure durations using paired *t* tests. At 80 ms, participants tended to report more correct letters when pairwise grouped with a connected line than when ungrouped, *t*(20) = 1.87, *p* = .08, whereas no such trend was observed at 200 ms, *t*(20) = −1.10, *p* = .29 (Figure 3b).[Fig-anchor fig3]

To examine the effect of perceptual grouping on attentional selection, we estimated the mean selectivity (α) for the three different grouping conditions (see Table 2). α differed significantly across conditions, *F*(2, 40) = 8.6, *p* = .001. Compared with the condition where the characters were not grouped, the selectivity of the participants for targets relative to distracters was increased by grouping the stimuli along the task-relevant dimension (*p* < .001), but not when each target was grouped with a distracter by a connected line (*p* = .91). Converging results were obtained when analyzing the number of correctly reported letters in the partial report trials using a repeated measures ANOVA with exposure duration (80 ms, 200 ms) and grouping condition (no grouping, task-relevant grouping, and task-irrelevant grouping) as within-subjects factors (Figure 3c). The main effects of grouping, *F*(2, 19) = 16.47, *p* < .001 and exposure duration, *F*(1, 20) = 388.32, *p* < .001 were significant, but not the two-way interaction, *F*(2, 19) = .03, *p* = .97.

**Figure 4 fig4:**
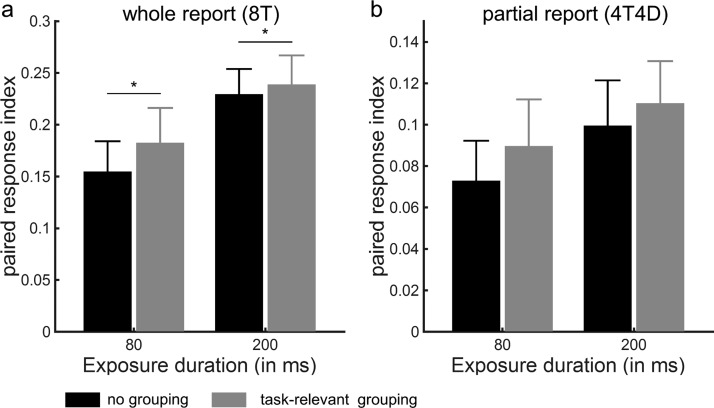
Experiment 1: paired response index. Paired response index in the whole report (b) and partial report (c) trials, as a function of exposure duration and grouping condition. The paired response index reflects the average number of correctly reported pairs of letters, corrected for what would be expected by chance. Error bars represent *SEM*.

We then examined the effect of perceptual grouping on the spatial configuration of the attentional priority map by calculating the PRI indicating participants’ tendency to report pairs of connected relative to separate letters. When eight targets were presented without distracters, we observed main effects of grouping, *F*(1, 20) = 5.41, *p* = .03 and exposure duration, *F*(1, 20) = 13.94, *p* = .001 but no two-way interaction, *F*(1, 20) = 2.02, *p* = .17. Even though grouping did not significantly increase the number of correctly reported letters (Figure 3b), participants were more likely to report two letters belonging to a pair along the horizontal or vertical axis when the letters in a pair were grouped through a connected line (Figure 4a). When distractors were present to the display (4T4D), we observed a main effect of exposure duration, *F*(1, 20) = 9.81, *p* = .005, but no significant main effect of grouping, *F*(1, 20) = 2.03, *p* = .17 and no significant two-way interaction, *F*(1, 20) = 0.11, *p* = .75.[Fig-anchor fig4]

In summary, we found that embedding elements into larger objects by means of uniform connectedness influenced distinct aspects of the attentional priority map but only when grouping occurred between elements of similar task relevance: Task-relevant grouping increased top-down control and influenced which elements were encoded in VSTM and available for explicit report. Contrary to our hypothesis, grouping each target with a distracter did not have a detrimental effect on performance.

## Experiment 2: Target Selection or Distracter Rejection?

In the second experiment, we investigated whether the effect of task-relevant grouping on distinct aspects of the attentional priority map (parameter α and PRI) observed in Experiment 1 was because of targets being encoded as groups rather than as single letters (higher attentional weight for targets), because of distracters being rejected as groups rather than as single letters (lower attentional weight for distracters), or both. To this end, we independently manipulated pairwise grouping of targets versus distracters.

### Method

#### Experimental conditions

Six different stimulus displays were realized, with either only targets being presented (8T, whole report) or targets and distracters being presented (4T4D, partial report). The first four conditions (8T no grouping, 8T grouping, 4T4D no grouping, and 4T4D task-relevant grouping) were identical to the ones used in Experiment 1. We collected 48 trials for each condition that was also present in Experiment 1 (Stimulus Display × Exposure Duration). Two new partial report conditions were introduced (Figure 1c). In the 4T4D target-target grouping, the four targets were pairwise grouped but the distracters remained ungrouped. In the 4T4D distracter-distracter grouping, the four distracters were pairwise grouped but the targets remained ungrouped. We collected 96 trials for each of the four new conditions (Stimulus Display × Exposure Duration). The order of the conditions was randomized for each subject. The task consisted of one practice block of 24 trials, followed by 16 blocks of 60 trials (i.e., 960 trials in total).

#### Data analysis

The procedure for data analysis was similar to the one of Experiment 1, with the only difference being that four different grouping conditions were considered in the analysis. Accordingly, four different selectivity parameters were used to model the data in Experiment 2 using TVA, one for each of the four grouping conditions. The numbers of correctly reported letters in the whole and partial report trials were submitted to repeated measures ANOVAs with exposure duration and grouping condition as factors. When sphericity could not be assumed (Mauchly’s sphericity test: *p* < .05), *p* values were adjusted using the Greenhouse-Geisser correction. Post hoc comparisons included pairwise comparisons between different grouping conditions (threshold: *p* < .05, Bonferroni-corrected for multiple comparisons; corrected thresholds whole report trials: *p* < .0125 for the interaction between grouping condition and exposure duration; corrected thresholds partial report trials: *p* < .0125 for the main effect of grouping condition and *p* < .006 for the interaction between grouping condition and exposure duration).

### Results and Discussion

**Figure 5 fig5:**
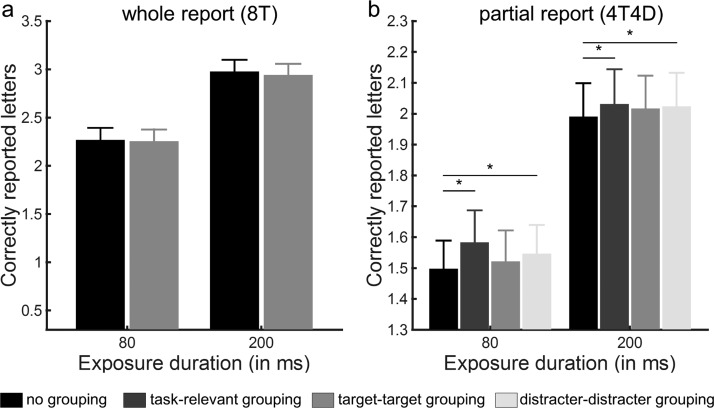
Experiment 2: correctly reported letters. Mean number of correctly reported letters in the whole report (a) and partial report (b) trials, as a function of exposure duration and grouping condition. Error bars represent *SEM*.

**Figure 6 fig6:**
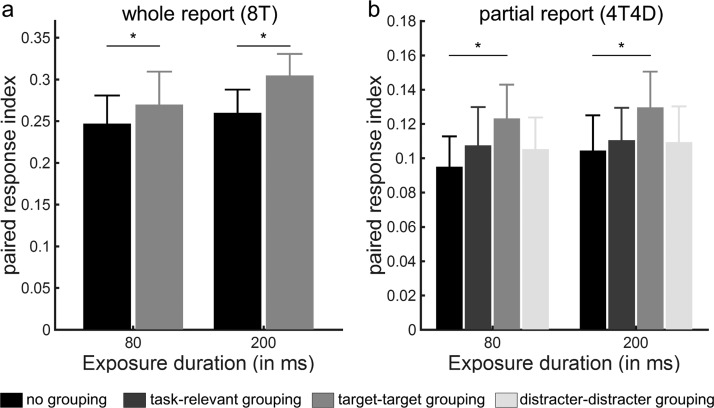
Experiment 2: paired response index. Paired response index in the whole report (a) and partial report (b) trials, as a function of exposure duration and grouping condition. The paired response index reflects the average number of correctly reported pairs of letters, corrected for what would be expected by chance. Error bars represent *SEM*.

We replicated the whole report findings obtained in Experiment 1 in an independent sample of 22 volunteers (Tables 1 and 2, Figure 5). A repeated measures ANOVA on the mean number of correctly reported letters in whole report trials with exposure duration (80 ms, 200 ms) and grouping condition (no grouping, task-relevant grouping) as factors revealed a main effect of exposure duration, *F*(1, 21) = 240.75, *p* < .001, but no main effect of grouping, *F*(1, 21) = 1.12, *p* = .30 and no significant interaction, *F*(1, 21) = 0.17, *p* = .61 (Figure 5a). In contrast, when analyzing the PRI, we observed a main effect of grouping, *F*(1, 21) = 8.42, *p* = .009, but no main effect of exposure duration, *F*(1, 21) = 1.15, *p* = .30 and no two-way interaction, *F*(1, 21) = 0.40, *p* = .54 (Figure 6a). [Fig-anchor fig5][Fig-anchor fig6]

The TVA-based model with four α-values was a good fit for the observed data (Tables 1 and 2). The Pearson product–moment correlation coefficient between the observed and predicted scores was on average 0.98 (*SD*: 0.02); thus, the model explained on average 95% (*r*^2^) of the variance in the data.

α-values differed significantly across conditions, *F*(3, 63) = 4.09, *p* = .01. Compared with the condition where the characters were not grouped, the selectivity of the participants for targets relative to distracters was increased by grouping the stimuli along the task-relevant dimension (*p* = .01). There was a similar trend when only distracters were grouped and targets remained ungrouped (*p* = .02), but not when only targets were grouped and distracters remained ungrouped (*p* = .18). Converging results were obtained when analyzing the number of correctly reported letters using a repeated measures ANOVA with exposure duration (80 ms, 200 ms) and grouping condition (no grouping, task-relevant grouping, target-target grouping, and distracter-distracter grouping) as within-subjects factors. The main effects of grouping (*F*(2.08, 43.72) = 5.34, G-G adj. *p* = .007 and exposure duration, *F*(1, 19) = 170.16, *p* < .001 were significant, but not the two-way interaction, *F*(3, 63) = 0.70, *p* = .56. Compared with the no grouping condition, the number of correctly reported letters was higher when characters were grouped along the task-relevant dimension (*p* = .005) or when only distracters were grouped (*p* = .006), but not when only targets were grouped and distracters were not (*p* = .10; Figure 5b).

A repeated measures ANOVA on the PRI revealed a main effect of grouping, *F*(3, 63) = 4.43, *p* = .005, but no main effect of exposure duration, *F*(1, 21) = .62, *p* = .21 and no two-way interaction, *F*(3, 63) = .07, *p* = .97. Post hoc paired *t* tests showed that, compared with the no grouping condition, only target-target grouping increased the PRI significantly (*p* = .001; Figure 6b).

In summary, the data of Experiment 2 suggest that the effect of task-relevant grouping on top-down control (parameter α) observed in Experiment 1 can be attributed to distracters being rejected as groups (lower attentional weight for distracters) rather than to targets being encoded as groups (higher attentional weight for targets). In addition, we replicated the observation that pairwise grouping of targets increases the tendency of participants to report grouped compared with separate letters.

## Summary and Concluding Discussion

We investigated how object-based perceptual representations interact with feature-based pertinence values to shape distinct aspects of the attentional priority map: the spatial distribution of attentional weights and top-down control, the difference in attentional weights between targets and distracters. In a whole or partial report task, we found that grouping characters along the task-relevant dimension affected performance in two independent ways: (a) pairwise grouping of targets changed the topography of the attentional priority map—thereby determining which of the targets were selected and available for explicit report, and (b) pairwise grouping of distracters increased the number of correctly identified targets by reducing the attentional weights of the distracters. Contrary to our hypothesis, pairwise grouping of each target with a distracter did not affect performance.

Our study differs in several ways from previous paradigms showing object-based effects (Chen, 2012). First, our study is the first to assess object-based effects in a multielement display, and their dependency on the behavioral importance of the object elements. Our experimental design also allowed us to assess how the configuration of elements into larger objects, which in themselves where irrelevant to the task, changed the topography of the attentional priority map. We used the unified framework of TVA to parametrize for each participant top-down control, that is, the tendency to assign higher attentional weights to targets relative to distracters (α), and advanced mathematical analysis of the distribution of the correctly reported letters across the visual field (PRI) to assess the spatial distribution of attentional weights.

Perceptual pairwise grouping of multiple targets by uniform connectedness significantly altered the spatial distribution of the attentional priority map, increasing the tendency of participants to jointly report or jointly miss elements that belonged to the same object (Dent et al., 2011; Gilchrist, Humphreys, Riddoch, & Neumann, 1997; Kahneman & Henik, 1981). In our study, exposure durations of 80 ms were sufficient to establish robust object-based representations and elicit object-based effects. This is surprising, given that some previous studies have shown less reliable object-based effects with short relative to long exposure durations (e.g., Avrahami, 1999; Chen & Cave, 2008; Law & Abrams, 2002; but see Duncan, 1984). Chen (2012) pointed out that the quality of the object-based representation may be more important than the specific exposure duration. We indeed maximized the goodness of object-based representations by using the principle of uniform connectedness (Kramer & Jacobson, 1991), with objects having closed rather than open boundaries (Posner et al., 1982) and object elements being presented on the same straight line within the object rather than separated by an angle or corner (Kravitz & Behrmann, 2011). Further studies should investigate the validity of our results when elements are grouped via other Gestalt laws.

Pairwise grouping of targets did not increase the number of targets that could be reported (whole report condition in Experiments 1 and 2, target-target grouping in Experiment 2). Our results are, therefore, only partly in alignment with previous studies in healthy volunteers showing facilitated responses to multiple (most of the time only 2) targets when they group to form an object (e.g., Behrmann et al., 1998; Duncan, 1984). In our study, the number of targets presented on each trial exceeded the capacity of VSTM, both in the grouping and in the no-grouping condition. The absence of object-based effects in whole-report trials was not surprising, under the assumption that the capacity of VSTM was constant across conditions. Furthermore, the targets remained individual elements that had to be identified within each perceptual group. Therefore, it is likely that two letters, albeit connected, were not encoded as different “features” of a single object in VSTM but occupied different slots in memory. Another possible explanation is that participants attended one object, and attention spread throughout the perceptual group, but that there was then a cost from shifting attention across object (Brown & Denney, 2007; Lamy & Egeth, 2002). However our data do not support that hypothesis, as most participants were able to report around three letters at 200 ms in the whole report trials and this did not depend on the presence of object-based representations.

Our second main finding is that pairwise grouping of distracters increased the number of correctly reported letters. These results are consistent with previous studies showing facilitated visual search when distracters are grouped such that they can be rejected together (Dent et al., 2011; Donnelly, Humphreys, & Riddoch, 1991; Gilchrist et al., 1997). This has been linked to a process of spreading suppression (Duncan & Humphreys, 1989). Previous studies that have attempted to assess spreading suppression directly have used probe detection tasks where it has been found that probes are more difficult to detect when they fall on distracters or carry a feature of the rejected distracters (Baithwaite, Humphreys, & Hulleman, 2005; Dent et al., 2011). Here we demonstrated evidence for spreading suppression directly through parameter estimates within the TVA framework. Our TVA modeling showed that pairwise grouping of distracters suppressed the attentional weight of distracters relative to targets; therefore, increasing the selectivity for targets.

Unlike previous studies showing that grouping targets with distracters is detrimental for performance (Driver & Baylis, 1989; Scholl, Pylyshyn, & Feldman, 2001), we did not observe significantly affected performance in this condition. In other words, in our study top-down control was not altered by pairwise grouping each target with a distracter. One possibility is that attentional weights do not spread when the target and distracter are not perceived as a single object (Richard et al., 2008). As letters are often read together in everyday life, as is the case with digits, the spreading of attentional weights might be facilitated when letters are grouped with letters and when digits are grouped with digits. These configurations, according to alphanumerical class, are highly familiar to the readers in contrast to objects containing letters intermixed with digits. Previous studies indeed suggest that attention is preferentially allocated to familiar shapes (Vecera & Farah, 1997). Further studies should investigate whether similar results are obtained when task-relevant grouping is not confounded by familiarity, for example, by distinguishing targets and distracters by color. Second, in contrast to previous focused attention paradigms, we used displays containing at least four targets and participants made unspeeded responses so that our estimation of attentional weights was not confounded by complex motor demands. The use of four distinct targets, each appearing in different objects distributed across the visual scene, may have led to more complex higher-order interactions in the computation of attentional weights. Although our data confirm that spreading of attentional weights is not *automatic* (see also Freeman et al., 2014), further studies are necessary to identify its boundaries when participants are confronted with a complex visual scene.

To conclude, our data show that object representations interact with the pertinence values of the elements’ features and spatial locations to shape the attentional priority map. Our investigations suggest that TVA is a useful framework to quantify the effect of perceptual grouping processes on attentional selection, enabling, for example, a direct comparison of the strength of different grouping principles.

## Figures and Tables

**Table 1 tbl1:** Attentional Performance

Experiment	TVA parameters					
	*t*_*0*_		*C*		*K*	
	*M*	*SD*	*M*	*SD*	*M*	*SD*
Experiment 1	2	3	57	14	3.05	.53
Experiment 2	2	4	50	20	2.89	.53
*Note.* Units for the individual parameters are: *t*_*0*_ (ms), *C* (letters/second), and *K* (letters). TVA = Theory of Visual Attention.						

**Table 2 tbl2:** Top-Down Selectivity as a Function of Grouping Condition

Grouping condition	α	
	*M*	*SD*
Experiment 1		
No grouping	.54	.17
Task-relevant	.45	.14
Task-irrelevant	.54	.11
Experiment 2		
No grouping	.41	.24
Task-relevant	.35	.21
Target-target	.38	.21
Distracter-distracter	.36	.20
*Note.* α ranges from perfect selection at 0 and nonselectivity at 1.		

## Footnotes

1 *K* was assumed to vary on a trial-by-trial basis. The *K* value reported is the expected *K* given a particular probability distribution (i.e., the probabilities that on a given trial *K* = 1, 2, . . . , 8; Dyrholm et al., 2011).
